# An optimized live bacterial delivery vehicle safely and efficaciously delivers bacterially transcribed therapeutic nucleic acids

**DOI:** 10.1002/elsc.202200037

**Published:** 2023-02-05

**Authors:** Darcy S. O. Mora, Madeline Cox, Forgivemore Magunda, Ashley B. Williams, Lyndsey Linke

**Affiliations:** ^1^ SiVEC Biotechnologies Fort Collins Colorado USA; ^2^ Department of Microbiology, Immunology and Pathology Colorado State University Fort Collins Colorado USA

**Keywords:** antiviral, delivery platform, drug delivery, nucleic acid delivery, RNA interference

## Abstract

There is an unmet need for delivery platforms that realize the full potential of next‐generation nucleic acid therapeutics. The in vivo usefulness of current delivery systems is limited by numerous weaknesses, including poor targeting specificity, inefficient access to target cell cytoplasm, immune activation, off‐target effects, small therapeutic windows, limited genetic encoding and cargo capacity, and manufacturing challenges. Here we characterize the safety and efficacy of a delivery platform comprising engineered live, tissue‐targeting, non‐pathogenic bacteria (*Escherichia coli* SVC1) for intracellular cargo delivery. SVC1 bacteria are engineered to specifically bind to epithelial cells via a surface‐expressed targeting ligand, to allow escape of their cargo from the phagosome, and to have minimal immunogenicity. We describe SVC1's ability to deliver short hairpin RNA (shRNA), localized SVC1 administration to various tissues, and its minimal immunogenicity. To validate the therapeutic potential of SVC1, we used it to deliver influenza‐targeting antiviral shRNAs to respiratory tissues in vivo. These data are the first to establish the safety and efficacy of this bacteria‐based delivery platform for use in multiple tissue types and as an antiviral in the mammalian respiratory tract. We expect that this optimized delivery platform will enable a variety of advanced therapeutic approaches.

**Abbreviations**
CFU: colony forming units
DAP: diaminopimelic acid
DPI: day post infection
EID_50_
: 50% egg infective dose
HGT: horizontal gene transfer
IAV: influenza A virus
IM: intramuscular
IN: intranasal
*inv*
: *Yersinia pseudotuberculosis* invasin gene
LLO: Listeriolysin O
NP: Influenza A nucleoprotein
PA: Influenza A polymerase acidic protein
RNAi: RNA interference
shRNA: short hairpin RNA
siRNA: small (short) interfering/silencing RNA
SVC1: strain identifier for the *Escherichia coli* strain described in this study

## 1 INTRODUCTION

Nucleic acid and protein moieties have rapidly come to the forefront of biomedical research as alternatives to small molecules and other drugs for the treatment of a variety of human diseases as well as a powerful approach for developing next‐generation vaccines. To fully realize the potential of these therapeutic and vaccination approaches, proteins, and nucleic acids must be delivered to target cells. Currently, such delivery in patients is mostly limited to viral vectors and other chemical delivery modalities (e.g., lipid nanoparticles); however, the power of these approaches is limited by a number of drawbacks, including safety concerns, production cost, payload size limitations, immunogenicity, and cytotoxicity [1, 2, 3, 4, 5]. To overcome these limitations, alternative delivery systems must be developed.

For over three decades, there has been increasing interest in the use of engineered commensal or probiotic bacteria as an in vivo nucleic acid and protein delivery modality and, as part of the normal human flora, *Escherichia coli* has garnered particular interest [6, 7, 8, 9, 10, 11, 12, 13]. Bacteria represent a powerful and unique approach for the delivery of therapeutic moieties that can overcome many of the limitations of viral or chemical delivery systems. In most cases, bacterial systems are designed to deliver therapeutic moieties extracellularly, for example, into a tumor microenvironment, and targeting is achieved via passive effects, for example, physiological attraction to conditions in the target niche, for example, low oxygen levels in the tumor microenvironment. Less progress has been made in the use of bacteria for intracellular, that is, cytoplasmic, delivery. Other invasive bacteria engineered for nucleic acid delivery have been reported [6, 7, 8, 9], but this is the first report of invasive bacteria being used as an antiviral in a mammalian model.

To serve as a viable in vivo intracellular delivery approach, an ideal bacteria‐based delivery system should possess three key activities: (i) the bacterial cells must be able to specifically target a disease‐relevant cell type (e.g., epithelial cells); (ii) the bacteria must be able to enter the target cell upon arrival; and (iii) the bacteria must be able to escape the phagosome to deposit their cargo into the cytoplasm. While professional phagocytic cells (e.g., neutrophils) readily take up bacteria via phagocytosis [14], other cell types, which are clear front runners as therapeutic targets (e.g., epithelial cells), do not have intrinsic phagocytic activity; thus, the bacteria must be engineered to enable cellular uptake.

**PRACTICAL APPLICATION**

The safety and efficacy of an optimized live bacterial delivery platform for the production and delivery of therapeutic nucleic acids to mucosal epithelial cells is presented.

In the *E. coli*‐based system described here, the *E. coli* strain SVC1 has been genetically modified to meet these needs [9]. First, the SVC1 bacteria carry a heterologous gene encoding the *Yersinia pseudotuberculosis* invasin (inv) protein to allow uptake by the targeted eukaryotic cells. Invasin binds to b1 integrin [15, 16, 17, 18], which occurs with several integrin receptors (a3, a4, a5, a6, and av) [19]. The specificity for b1 integrin allows the bacteria to target only b1‐expressing cells [15]. Important for the usefulness of an invasin‐based targeting system is that b1 integrin is expressed by a wide variety of cells, including a broad group of epithelial cells, for example, intestinal [20], respiratory [21], eye [22], and others [19], as a well as certain types of cancer cells [23, 24]. Through local or systemic administration, the bacterial delivery vehicle can target various organs and tissues after which it interacts with specific types of epithelial or cancerous cells. Upon binding to b1 integrin on the target cell surface, the bacteria are taken up via receptor‐mediated phagocytosis, thereafter finding themselves compartmentalized within phagosomes.

For the cargo to reach the cytoplasm of the target cell, the cargo must be released from both the bacterial cell and the target cell's phagosome. These steps are enabled by two additional modifications to the SVC1 bacteria. First, the bacteria are auxotrophic for diaminopimelic acid (DAP) due to a null mutation in *dapA*, which encodes a protein essential for DAP biosynthesis; thus, DAP must be provided exogenously in the growth medium. As DAP is required for peptidoglycan crosslinking in the Gram‐negative cell wall, bacteria are structurally compromised in the absence of DA [13, 25]. Eukaryotic cells do not produce DAP, so upon entry the bacterial cells (i) cannot replicate since they cannot synthesize new cell wall and (ii) are sufficiently unstable that they rapidly lyse to release their cargo into the phagosome. Upon lysis in the phagosome, the bacteria release the product of a second heterologous gene, *hlyA*. This gene encodes the hemolysin HlyA from *Listeria monocytogenes* (also known as listeriolysin O or LLO) [26]. The liberated LLO protein perforates the endosomal membrane, thereby allowing the bacterial cargo to enter the cytoplasm of the eukaryotic cell [13, 27, 28]. The combined traits conferred by these genetic modifications render these bacteria useful as an efficient intracellular delivery vehicle.

An important consideration of any drug delivery vehicle that will be used in therapeutic applications is safety. Of particular relevance to bacterial drug delivery vehicles are the safety aspects of host colonization and immunogenicity. The parental strain of SVC1 has been highly domesticated since its isolation from a human patient in 1922 [29]. The lipopolysaccharide core on the outer membrane is defective in attachment of the O‐antigen, making SVC1 a rough strain. Furthermore, the cells do not express a capsular (K) antigen, further inhibiting colonization [25]. As mentioned above, the bacteria described here are attenuated via engineered DAP auxotrophy that, in addition to promoting cargo release in the phagosome, also acts as a powerful biocontainment mechanism, both in the patient and in the environment [25]. Furthermore, outside of a very low level of DAP in the urine (likely originating from lysed bacteria in the excretory system) [30], DAP is not otherwise known to exist in the human body or inside cells. Thus, SVC1 bacteria cannot replicate in patient tissues or within cells. Another additional safety concern is horizontal gene transfer (HGT), whereby genes that confer increased fitness under environmental conditions (e.g., antibiotic resistance genes) are passed from a donor strain to a compatible recipient strain [31]. HGT is mediated by a variety of plasmids and phages; importantly, the strain described here lacks such plasmids and phage [32], preventing it from disseminating cargo‐encoding recombinant molecules.

Current delivery systems, including lipid nanoparticles and viral vectors, suffer from a variety of immunological issues [33, 34, 35, 36, 37, 38, 39, 40] and the outcomes of undesirable immune responses can have devastating clinical repercussions, especially in applications requiring repeat administration. Therefore, the immunogenicity of any novel delivery system must be carefully characterized to mitigate such issues. As part of the normal flora, *E. coli* represents an appealing candidate for use as a bacteria‐based delivery vehicle, particularly in light of immunogenicity concerns.

There has been an ongoing interest in the use of RNA interference (RNAi) as a therapeutic modality in clinical applications [43, 44, 45]. The mechanism underlying RNAi therapy depends on intracellular delivery of an RNA molecule to a target cell that can then be processed by the cellular RNAi pathway to ultimately silence the expression of a specifically targeted pathological gene. RNAi‐mediated silencing is guided by small interfering RNAs (siRNAs) [46, 47, 48]. The SVC1 bacteria used in the antiviral described here express shRNAs that, upon delivery into the target cell cytoplasm, are rapidly processed into siRNAs by the eukaryotic RNAi machinery (Dicer). Importantly, bacteria, including *E. coli*, can be reliably engineered to express shRNAs targeting genes of interest [9, 28]. In the case of invasive strains, for example, SVC1, the bacteria can both produce and deliver the shRNAs to the cytoplasm of targeted cells to achieve desired therapeutic effects. Some progress has been made in the therapeutic use of shRNA‐expressing *E. coli* strains. For example, Cequent Pharmaceuticals created a therapeutic *E. coli* strain to treat familial adenomatous polypopsis, a disease of the gastrointestinal tract, by silencing endogenous beta‐catenin expression in gastrointestinal epithelial cells [28]. Preclinical and human clinical testing of this strain demonstrated its safety and efficacy, reinforcing the potential of *E. coli‐*mediated nucleic acid delivery for development in other clinical applications [10, 11, 49]. In another early application, Linke et al. [9] developed an shRNA‐based approach for the prophylaxis and treatment of avian influenza virus. In this system, two *E. coli* strains were engineered to independently silence the expression of two essential influenza viral proteins: an RNA polymerase subunit (polymerase acidic protein; PA) and a capsid protein (nucleoprotein; NP). When administered together to the respiratory tract, these bacterial strains can target most known influenza A strains while minimizing the development of therapeutic resistance, especially that resulting from genetic drift [50, 51, 52, 53]. The safety and efficacy of this approach were robustly validated in a chicken model of avian influenza [9], demonstrating that *E. coli‐*mediated nucleic acid delivery can indeed be used to mitigate the replication and shedding of a clinically important virus.

Bacterial delivery systems are gaining attention as a new approach for drug delivery; however, many questions remain about their safety. In this study, we have for the first time established our engineered strain, *E. coli* SVC1, as a safe and viable system for in vivo applications, particularly via validation in a murine model. Using an in vitro cell model, we first demonstrated that the SVC1 system can produce and deliver functional shRNA, resulting in RNAi gene silencing. We then examined the feasibility of directly administering SVC1 to specific tissues and organs and assessed its biodistribution in clinically relevant sites in vivo. Next, we investigated the safety profile of SVC1 upon repeated dosing to the lungs via histological analysis and monitoring of expression level changes in innate and adaptive immunity‐related genes. Finally, we validated the ability of SVC1 to deliver therapeutic shRNAs, functioning as an antiviral, using an in vivo murine influenza virus infection model. Taken together, the results presented here support the potential of SVC1 to serve as a powerful delivery platform for therapeutic nucleic acids and support translational research to drive its future clinical development.

## 2 MATERIALS AND METHODS

### 2.1 Bacterial strains, plasmids, and cells

The invasive *E. coli* strain SVC1 is a K‐12 derivative [F^–^ *endA1 hsdR17 (rK^–^ mK^+^) glnV44 thi‐1 relA1 rfbD1 spoT1* Δ*rnc* Δ*dapA*]. The cells are auxotrophic for diaminopimelic acid (DAP) due to a deletion of *dapA. E. coli* cells were cultured in brain–heart infusion medium supplemented with DAP (100 μg/mL) and appropriate antibiotics at the following concentrations: kanamycin, 25 μg/mL; ampicillin, 100 μg/mL. A549 cells are a human adenocarcinoma alveolar basal epithelial cell line. For the in vitro GFP silencing experiments, an A549 cell line constitutively expressing GFP (Cell Biolabs, San Diego, California, USA, AKR‐209) was used. The identity of the A549/GFP cells was confirmed by GFP expression, morphology, and trypan‐blue dye exclusion, and all cell cultures were routinely monitored for microbial contamination using standard techniques. The construction of pSiVEC‐scramble (non‐specific small RNA sequence), pSiVEC‐PA, and pSiVEC‐NP, which were derived from pmbv43 [8], is described in a previous publication [9]. pSiVEC‐GFP was constructed using the DNA template encoding the shRNA specific for GFP [77] from the copepod *Pontellina plumata*: sense, GCTACGGCTTCTACCACTTT and antisense, AAAGTGGTAGAAGCCGTAGC. Using standard cloning and transformation methods, resulting SVC1 colonies transformed with the plasmids pSiVEC‐scramble, pSiVEC‐PA, pSiVEC‐NP, and pSiVEC‐GFP were screened by PCR, and a single positive clone was sequence validated and propagated. Stocks were generated and stored at −80°C in 20% glycerol. A single frozen aliquot from each construct stock was thawed to determine colony forming units (CFU)/mL via plate enumeration [9]. These strains are referred to as SVC1‐scramble, SVC1‐PA, SVC1‐NP, and SVC1‐GFP.

### 2.2 In vitro invasion assay

**FIGURE 1:**
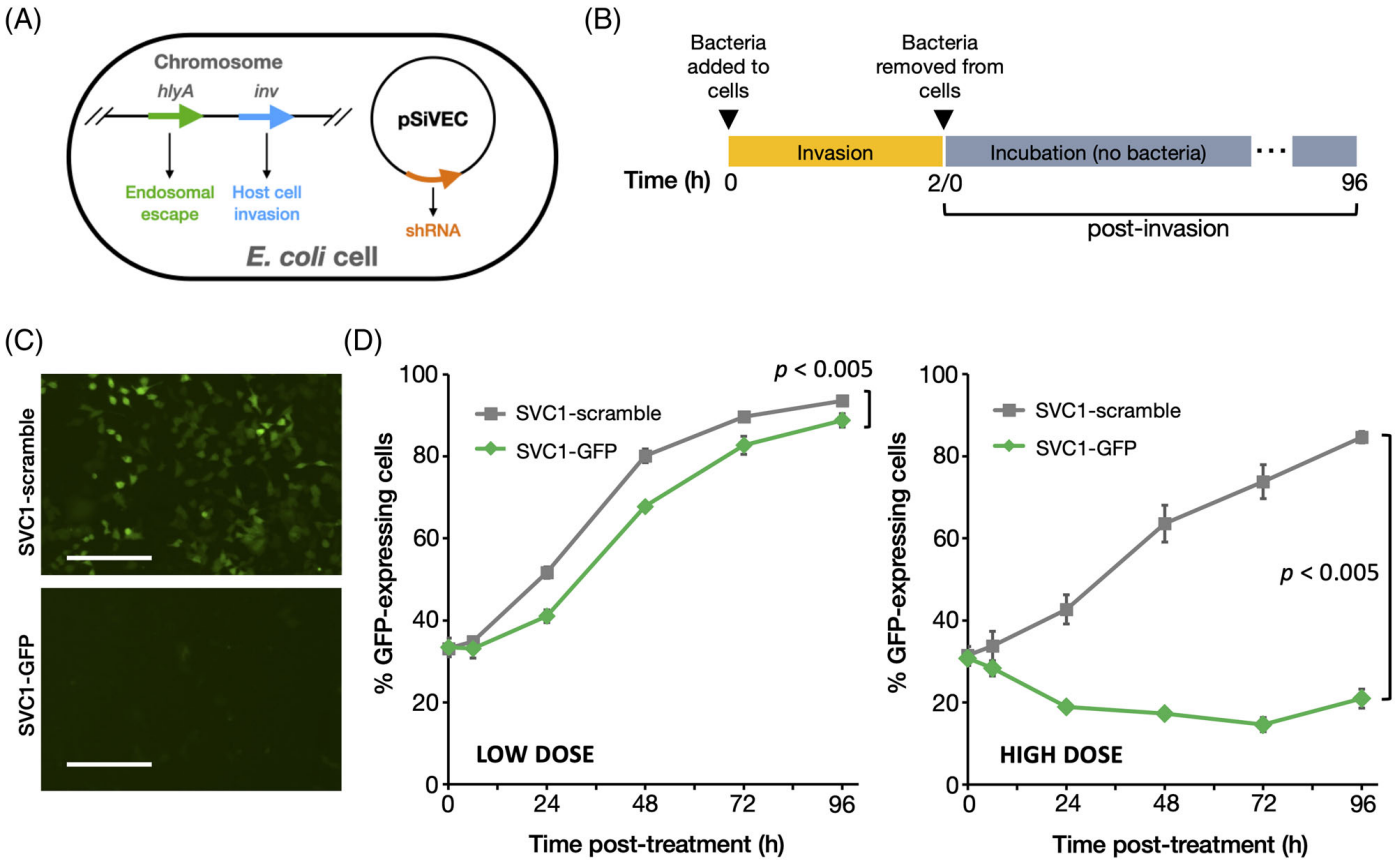
Bacterial delivery of shRNA‐GFP silences GFP expression in respiratory epithelial cells. (A) Genetic features of *E. coli* SVC1 bacteria. (**B**) Experimental design for shRNA delivery. A549 cells constitutively expressing a GFP reporter gene were seeded in 24‐well culture plates and incubated with two concentrations of bacteria (low dose, 1.56 × 10^6^ CFU/mL; high dose, 1 × 10^8^ CFU/mL) expressing a scramble (non‐targeting) small RNA or an shRNA targeting GFP, and GFP expression was monitored for 96 h post‐invasion. (C) Fluorescent image demonstrating GFP knockdown achieved via SVC1‐mediated GFP‐shRNA delivery (96 h post‐invasion; scale bar represents 0.5 mm). Overlay images (GFP and brightfield) are provided in Figure S2. (**D**) GFP knockdown reported as the percentage of GFP‐positive A549 cells. Plots show the mean ± SD at each time point. The statistical significance of the differences between the control and experimental groups was assessed using two‐way ANOVA, and the *p*‐values are provided. All experiments in Figure 1 were independently repeated at least three times and representative results are shown

For the GFP gene silencing studies (Figure 1), A549/GFP cells were seeded one day prior to invasion in a 24‐well tissue culture‐treated microplate with black walls and clear bottom (Perkin Elmer, Waltham, Massachusetts, USA; VisiPlate 1450) to allow the monolayer to reach 70% confluence. On the day of invasion, 1‐mL frozen aliquots of SVC1‐GFP and SVC1‐scramble were thawed, centrifuged, re‐suspended, and diluted to low (1.56 × 10^6^ CFU/mL) and high (1 × 10^8^ CFU/mL) concentrations in DMEM supplemented with DAP (100 μg/mL). Bacteria concentrations were selected using data from experiments (4‐fold dilution series) intended to empirically determine the minimum dose where an effect on GFP expression was detectable and the maximum tolerated dose without causing cell loss or cytopathic effect (CPE). A549/GFP cells were washed to remove antibiotics and incubated with 500 μL of SVC1‐GFP or SVC1‐scramble at low and high doses at 37°C with 5% CO_2_. After 2 h incubation, A549/GFP cells were washed three times, and fresh DMEM with antibiotics was added. GFP signal was measured at 0, 6, 24, 48, 72, and 96 h post invasion using the Nexcelom Celigo (Lawrence, Massachusetts, USA) and reported as the percentage of GFP‐positive A549 cells. The statistical significance of the differences between the SVC1‐scramble control and SVC1‐GFP experimental groups was assessed using two‐way ANOVA (*p* < 0.005). For the invasion assay shown in Figure S1, the same approach was used by SVC1 bacteria transformed with an RFP‐expressing plasmid, pE2‐Crimson (Clonetech, Mountain View, California, USA).

### 2.3 In vivo biodistribution assays

**FIGURE 2:**
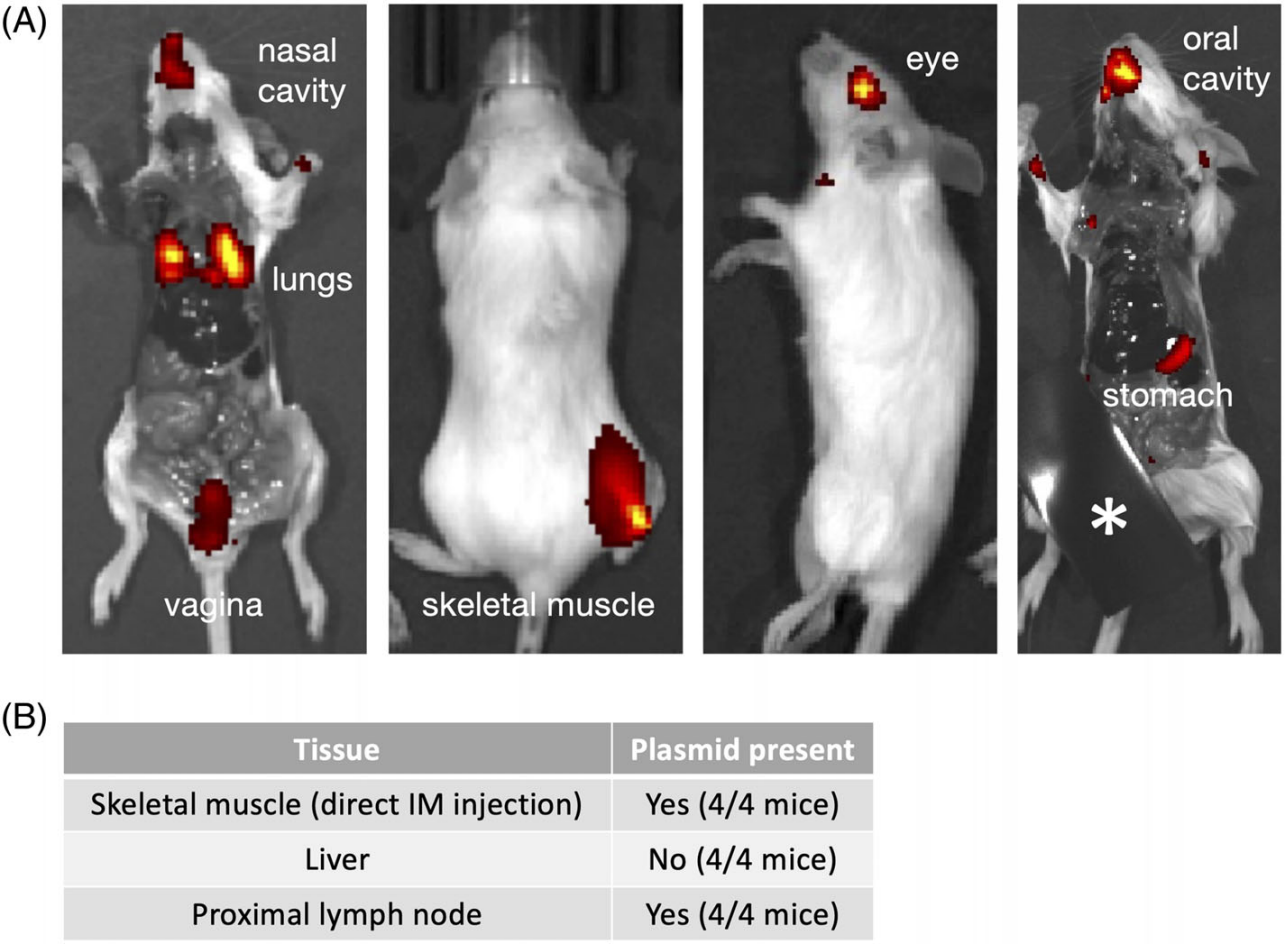
In vivo administration and clearance of invasive bacteria. (A) Imaging of fluorescently labeled SVC1 bacteria after delivery as described in the text and Table 1. The leg of the right‐most mouse is covered (see asterisk) to allow imaging of the weaker fluorescent signal in the stomach and oral cavity. Relative signal intensity is shown ranging from red (low) to yellow (high). (B) Assessment of plasmid presence in various tissues 72 h after IM delivery

All animal studies described were conducted according to the guidelines of the Declaration of Helsinki and approved by the Colorado State University Institutional Animal Care and Use Committee (IACUC), protocol no. 18–8110A. To characterize the biodistribution of the SiVEC vehicle following localized administration to mouse mucosal epithelial and skeletal muscle tissues (Figure 2A), SVC1‐scramble and isogenic non‐invasive SVC1 bacteria (lacking pSiVEC‐scramble) were fluorescently labeled using the XenoLight RediJect 750 near‐infrared fluorescent probe (Perkin Elmer, Waltham, Massachusetts, USA). Each dose contained 1 × 10^8^ or 1 × 10^9^ CFU, which was determined to be a sufficient dose to deliver enough fluorophore to the target tissues to be detected by the imaging system (i.e., 70 photons/s/sr/cm^2^). Nine‐week‐old female BALB/c mice (Jackson Laboratory, Bar Harbor, Maine, USA) were anesthetized with inhaled isoflurane in an anesthesia chamber and then transferred to the Perkin Elmer IVIS Spectrum system for in vivo imaging with an untreated control mouse included for calibration of background signal due to autofluorescence. Mice were treated with SVC1‐scramble (invasive) per the route of administration and dose per tissue type as shown in Table 1.

**TABLE 1:** Routes of administration of invasive SVC1 bacteria to various tissues

**Organ/tissue**		**Administration method**	**CFU (dose) per volume**
Eye		Eyedrop	1 × 10^9^ CFU/5 μL
Vagina		Vaginal instillation	1 × 10^9^ CFU/15 μL
Skeletal muscle		Intramuscular (IM) injection, thigh	1 × 10^8^ CFU/25 μL
Respiratory tract			
	Upper (nasal cavity)	Intranasal (IN)	1 × 10^9^ CFU/50 μL
	Lower (lungs)	IN droplet inhalation with anesthesia	1 × 10^9^ CFU/50 μL
Digestive tract			
	Oral cavity	Oral gavage	1 × 10^9^ CFU/100 μL
	Stomach	Oral gavage	1 × 10^9^ CFU/100 μL

To demonstrate localized delivery to mucosal epithelia (Figure 2A), mice were imaged at 0, 2, 4, 6, and 18 h post‐administration. Eighteen hours post‐treatment, the mice were euthanized, and the body cavity of each animal was opened with a ventral, longitudinal incision, extending from the vaginal opening, up through the lower jaw to expose deeper tissues difficult to image. One final image was captured of all mice.

To demonstrate injected delivery to the skeletal muscle (thigh) and to characterize the route of bacterial vehicle clearance (Figure 2B), mice were imaged at 0, 6, 20, 48, and 72 hours post‐IM injection. Seventy hours post‐IM injection, mice (*n* = 4) treated with SVC1‐scramble were euthanized, and liver, draining lymph tissue, and the thigh muscle were collected for subsequent DNA extraction to screen for the presence of SVC1‐scramble. Briefly, DNA was extracted from 20 mg of each tissue using the ZYMO Quick‐DNA Miniprep Plus Kit (Zymo Research, Irvine, California, USA). Resulting DNA was amplified neat and diluted 1:2, and 1:5 in molecular water with conventional PCR using primers specific to pSiVEC‐scramble: forward, CAGATGCGTAAGGAGAAAATACCGCAT; reverse, CATTAATGAATCGGCCAACGCGCG. PCR amplification was completed using a 25 μL reaction containing 5 μL DNA template, 12.5 μL 10X GoTaq G2 Hot Start Master Mix (Promega Corporation, Madison, Wisconsin, USA), and 1 μM final each primer. Cycling conditions consisted of 94°C for 4 min followed by 30 cycles of 94°C for 30 s, 56°C for 30 s, and 72°C for 2 min, and a final elongation step at 72°C for 10 min. PCR products were analyzed by 2% agarose gel electrophoresis. The limit of detection for DNA extraction and PCR amplification of SVC1‐scramble was 10^2^ CFU/20 mg tissue.

### 2.4 Immunogenicity assay and histopathology

**FIGURE 3:**
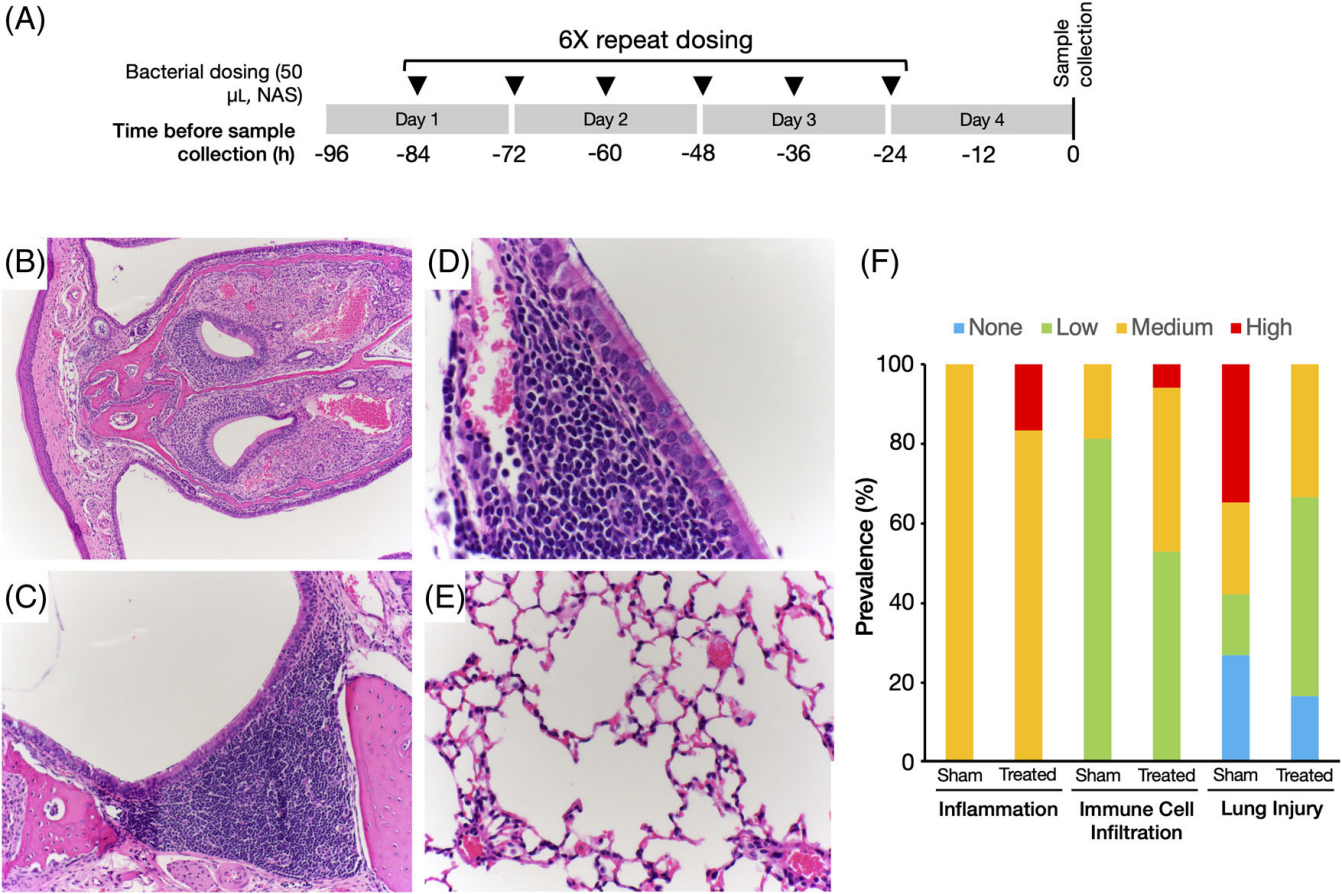
Repeat in vivo dosing of invasive bacteria to the lungs does not adversely affect tissue homeostasis. (A) Mice were anesthetized and dosed intranasally with invasive SVC1 bacteria (1 × 108 CFU per 50 μL dose, *n* = 5) in PBS (treated) or PBS alone (*n* = 5) (sham). Tissue samples were collected for analysis 24 h after administration of the last dose. (B) Cross‐section of the inferior nasal meatus with normal pseudostratified ciliated respiratory epithelium and the vomeronasal organ and glands, (C) close‐up of normal ciliated respiratory epithelium and underlying nasal mucosa‐associated lymphoid tissue, (**D**) nasopharyngeal duct and nasal‐associated lymphoid tissue, (**E**) normal lung tissue. (**F**) A summary of the prevalence of inflammation, immune cell infiltration, and injury in the lungs based on histological examination. The categories of low, medium, and high are defined in the Section 2

The safety of repeat dose administration to the lungs and the effect on tissue pathology and immune response was evaluated in 9‐week‐old female BALB/c mice. SVC1‐scramble (1 × 10^8^ CFU/50 μL) or a phosphate‐buffered saline (PBS)‐sham (50 μL) treatment was administered intranasally to *n* = 5 mice per group approximately every 12 h for six doses total (Figure 3A). Twenty‐four hours after administration of the last dose, mice were euthanized and dissected for gross pathology assessment and the collection of tissues for histopathology and gene expression profiling. The spleen was collected from each animal and stored in RNAlater (Thermo Fisher Scientific, Waltham, Massachusetts, USA) for subsequent RNA extraction and analysis using the Qiagen RT^2^ Profiler Mouse Innate and Adaptive Immune Response PCR Array (Qiagen, Hilden, Germany). The lungs were placed in a tissue cassette and fixed in 10 volumes of 10% neutral buffered formalin (NBF) for 24 h prior to submission to the histology division of the Colorado State University Veterinary Diagnostic Laboratory (CSU VDL). The head was severed, and the lower jaw, skin, and excess tissue, including cheek fat pads and muscle, were removed. The posterior/rostral two‐thirds of the skull and brain were removed, exposing the olfactory bulb, but leaving the skull on the lateral aspects of the head. This portion of the head containing the nasal cavity was immediately fixed in 10 volumes of 10% NBF for 24 h at room temperature. After 24 h, the fixed heads were rinsed 5× with deionized water, decalcified in 25 mL of 5% formic acid with moderate agitation for 24 h at room temperature, and rinsed 3× with 0.01 M PBS. The heads were then cut into three sections to expose the nasal cavity and nasal turbinate structure, arranged in a tissue cassette, and transferred back into 10% NBF to fully cover each cassette. The head samples were then submitted to the CSU VDL. Fixed lung and head samples were embedded in paraffin, serially sectioned, mounted, and stained with hematoxylin and eosin (H&E). Slides were pathologically evaluated using a 5‐point scale to score the degree of inflammation (fibrin, edema, vasculitis, bacteria presence), immune cell infiltration (neutrophils, lymphocytes, plasma cells, macrophages), and lung injury (bronchial epithelial hyperplasia, emphysema). The following scale was used to score each slide: 0: absent (“none”), 1: minimal (“low”), 2–3: mild to moderate (“medium”), and 4: severe (“high”).

Total RNA was extracted from 5 mg of spleen tissue using the Mag‐Bind Total RNA 96 kit (Omega Bio‐Tek, Norcross, Georgia, USA) and a KingFisher Flex Instrument (ThermoFisher Scientific, Waltham, Massachusetts, USA), and RNA concentration and purity were determined via Nanodrop (ThermoFisher Scientific) spectrometry. The Qiagen RT^2^ First Strand Kit was used to reverse‐transcribe 500 ng of each spleen RNA sample, per kit instructions. The resulting cDNA was stored at −20°C until analysis on the immune array. Expression of 84 immune‐related genes was analyzed by qPCR with the Qiagen Mouse Innate & Adaptive Immune Response RT^2^ Profiler PCR Array, per kit instructions. A single sample (representing each mouse) was assessed on each array plate on a Roche Light Cycler 480 II real time PCR thermal cycler (Roche, Basel, Switzerland). All data were analyzed with Qiagen's Gene Globe online analysis application. Gene expression was normalized to four housekeeping genes: beta‐actin, beta‐glucuronidase, heat shock protein 90 (alpha), and beta‐2 microglobulin. Gene expression changes were considered statistically significant at *p* > 0.05, while functionally significant differences were defined as a fold increase of ≥2. Results were independently reviewed by a qualified immunologist (subject matter expert) for assessment of a biologically relevant immune response to the SVC1 bacterial treatment.

### 2.5 In vivo IAV challenge assays

To demonstrate the therapeutic potential of the SiVEC delivery vehicle, SVC1‐PA and SVC1‐NP were constructed to express shRNAs targeting the influenza A viral PA and NP mRNAs, respectively, for delivery to the respiratory tissues in an established murine influenza disease model [78, 79, 80]. These strains were generated as previously described [9] and were mixed 1:2 (SVC1‐PA:SVC1‐NP) to create an antiviral cocktail referred to as SiVEC‐IAV. Eight‐week‐old female BALB/c mice (*n* = 96) were anesthetized with inhaled isoflurane and dosed with SiVEC‐IAV or PBS–sham by intranasal instillation. Mice were treated with 50 μL of PBS or SiVEC‐IAV in high (1 × 10^8^ CFU/mL) or low (1 × 10^6^ CFU/mL) doses twice prior to and four times after infection with 1 × 10^4^ EID_50_ per 25 μL dose of influenza A virus, A/Puerto Rico/89VMC3/1934 (H1N1) (BEI Resources, NIAID, NIH, NR‐29028) (see Figure 5A). Three, 5, 7, and 9 days post infection (DPI), mice (*n* = 6 per treatment/dose and time point) were euthanized, and nasal turbinates were collected and placed in RNALater.

H1N1 virus titers in the nasal turbinates of mice treated with the high and low SiVEC‐IAV doses were measured via RT‐qPCR and fold reductions in viral titer were calculated relative to the PBS‐sham control group. Briefly, total RNA was extracted from approximately 5 mg of nasal turbinate tissue using the Mag‐Bind Total RNA 96 RNA extraction kit with a KingFisher Flex purification system, and RNA concentration and purity was determined using Nanodrop spectrometry. RNA was diluted to 3 ng/μL and reverse transcribed and amplified using the Power SYBR Green RNA‐to‐CT 1‐Step Kit (Thermo Fisher Scientific) to detect the presence of influenza A matrix gene (M‐gene). Primer sequences were as follows: M‐gene forward, CTTCTAACCGAGGTCGAAACGTA, and M‐gene reverse, GGTGACAGGATTGGTCTTGTCTTTA. RT‐qPCR amplification was completed using a 20 μL reaction containing 5 μL RNA template, 10 μL 2× Power SYBR Green, 0.16 μL 125× RT enzyme, and 0.2 μM of each primer. The RT‐qPCR cycling conditions were 48°C for 30 min, 95°C for 10 min, 40 cycles of 95°C for 15 s, and 60°C for 1 min, followed by 60°C for 5 s and 95°C for 5 s to visualize the melting curve for each RT‐qPCR assay. The standard curve for virus quantification was generated in triplicate using a series of 10‐fold dilutions from 1 × 10^1^ to 1 × 10^7^ EID_50_ of the H1N1 stock virus from which the EID_50_ equivalent per mL (EID_50_ eq/mL) of each sample was calculated from Cq. The limit of detection was determined to be 10^1^ EID_50_/mL (1 log_10_ EID_50_/mL) per reaction. Statistical significance in fold reduction in viral titer between treated and PBS sham mice was calculated using the two‐sample Wilcoxon rank‐sum (Mann–Whitney *U*) test (*p* < 0.05).

## 3 RESULTS

### 3.1 Invasive SVC1 *E. coli* cells deliver functional shRNA in vitro

To assess the RNAi activity of shRNAs expressed from the SiVEC plasmid (pSiVEC) and delivered to eukaryotic cells by invasive, non‐pathogenic *E. coli* cells (SVC1), we measured green fluorescent protein (GFP) depletion in human alveolar basal epithelial cells (A549 cells) stably expressing GFP. We treated A549 cells with SVC1 carrying pSiVEC encoding a GFP shRNA (SVC1‐GFP) or pSiVEC encoding a non‐targeting small RNA (SVC1‐scramble) for 2 h. The ability to invade A549 cells was confirmed via a fluorescence‐based assay (Figure S1). Subsequently, we removed the bacteria, and the cells were further incubated for an additional 96 h. We measured GFP expression 6, 24, 48, 72, and 96 h after bacterial removal (Figure 1B). As shown in Figures 1C and S2, GFP expression was robustly reduced in A549 cells treated with SVC1‐GFP compared with A549 cells treated with SVC1‐scramble. GFP depletion persisted over 96 h at both a low dose (Figure 1D, left) and at a high dose of bacteria (Figure 1D, right). As would be expected, the higher dose of invasive bacteria resulted in more robust GFP depletion (i.e., a dose‐dependent response), suggesting that the level of depletion achieved via shRNA delivery can be controlled by varying the number of invasive bacteria. Importantly, treatment with these levels of bacteria did not lead to any increased CPE or cell death (see Figure S2). The length of the cell cycle of A549 cells under the growth conditions used here is approximately 20 h (Figure S3); thus, some cell division likely occurred over the course of this experiment. The observation that GFP expression did not increase during at least the first 72 h of the time course suggests that the abundance of delivered shRNA is sufficient to be inherited by the daughter cells of the originally invaded cells, which has been previously reported for siRNA [54]. Taken together, the data presented in Figure 1 confirm that SVC1 can invade eukaryotic epithelial cells and deliver a cargo of shRNA that is then processed via the RNAi pathway to robustly and persistently silence the expression of a target gene.

### 3.2 Invasive SVC1 *E. coli* can be administered to various epithelial tissue types

SVC1 binds to target cells via an interaction between its surface‐expressed invasin (Figure 1A) and β1 integrin on the surface of the target eukaryotic cells. β1 integrin is expressed by epithelial cells in multiple tissue types, including those in the cornea, respiratory tract, reproductive tract, digestive tract, and skeletal muscle. This ubiquity suggests that SVC1 can be used as a flexible approach for delivering therapeutic moieties to various tissues. To examine the versatility of SVC1 as a delivery system, we used various methods (Table 1) to apply invasive, fluorescently labeled SVC1 bacteria to the eye, upper (nasal cavity) and lower (lungs) respiratory tract, vagina, digestive tract, and skeletal muscle.

As shown in Figure 2A, SVC1 bacteria remained localized in the targeted organs and tissues following administration, suggesting that they can indeed be used for localized organ‐ and tissue‐specific delivery of therapeutic cargo without trafficking to other undesired sites in the body. These results validate the versatility of SVC1 for use as a delivery vehicle in various tissue types. Our ongoing work suggests that target cell invasion is efficient; nevertheless, our in vivo administration method likely introduces a surplus of bacteria. In light of this consideration, we were interested in the fate of any excess, that is, non‐phagocytosed bacterial cells. The clearance of the system is especially important with regard to trafficking of the SVC1 delivery vehicle to the liver, which is often undesirable in drug delivery applications due to associated toxicity [55, 56, 57]. To explore this feature of SVC1, we administered SVC1 intramuscularly in the hind limb and then collected a section of muscle tissue that received the localized injection, liver, and proximal draining lymph node after 20 and 72 h. We then tested for the presence of the delivery vehicle in the injected muscle as well as the liver and lymph nodes using PCR with primers that detect the SVC1‐borne plasmid (pSiVEC) in total DNA isolated from each tissue sample as shown in the table in Figure 2B. The PCR results suggest that the excess bacteria are cleared via the lymphatic system rather than being trafficked to the liver.

### 3.3 Repeat dosing of SVC1 to the respiratory tract is well tolerated in vivo

To explore the potential of SVC1 as a tissue‐targeting nucleic acid delivery platform, we focused on the respiratory tract. By virtue of their ability to silence expression of target proteins, therapeutic shRNAs have broad usefulness in the treatment of a variety of infectious diseases of the lungs, including respiratory viruses (e.g., influenza and SARS viruses); however, because of the intrinsic instability of shRNAs in the cytoplasm and the transience of the SVC1 bacteria at the delivery site (see above), a robust therapeutic effect would likely require repeat administration. Various issues with repeat dosing (e.g., undesirable immune responses and acquired resistance) limit the usefulness of some current delivery systems, especially adeno‐associated virus (AAV)‐based systems [33, 34, 35, 36, 37]; therefore, we were interested in whether SVC1 could overcome such limitations, especially detrimental immunogenicity. To this end, we examined the effects of repeat dosing of SVC1 to the respiratory tract. We administered six doses of 50 μL containing 1 × 10^8^ SVC1 bacteria suspended in sterile PBS intranasally to mice over the course of 60 h (Figure 3A). As a control, we also treated mice with PBS alone (sham). Twenty‐four hours after the sixth dose, we euthanized the mice and collected tissue samples for analysis. We monitored posture, grooming, interest in food, and behavior, and all were normal prior to euthanasia, and all of the SVC1‐treated mice appeared healthy. Upon dissection, we found no gross abnormalities or differences in the lungs, liver, kidneys, heart, abdominal cavity, nasal cavity, spleen, or other internal anatomy, and we did not observe any gross lesions (data not shown). Nasal sections from SVC1‐ and sham‐treated mice had normal morphology with no inflammation, no bacteria, no increase in mucus secretion, and no alteration in the mucociliary apparatus (representative nasal sections are shown in Figure 3B,C). We also examined the lungs for three pathologies: inflammation, immune cell infiltration, and injury (Figure 3D–F). The lung tissue samples all had minimal to mild increase in alveolar and intracapillary macrophages affecting predominantly one or two lobes that did not vary significantly among the SVC1‐ and sham‐treated animals, suggesting a baseline responsive mild lung infiltrate that was not distinctly due to the treatment nor made worse by the treatment. In rare cases, the blood vessels were minimally reactive and surrounded by edema and rare fibrin, supporting the possibility of a hematogenous antigen source unrelated to either treatment (SVC1 or sham). A single SVC1‐treated mouse had severe lymphoplasmacytic and histiocytic pneumonia that varied significantly from the other mice. There was subjectively a moderate lymphoid aggregate (BALT) hyperplasia, supporting a mild baseline immune response; however, this pathology was uniformly present and could not be ascribed to SVC1 bacterial treatment. The immunogenicity of SVC1 was then examined objectively as described in detail below. We did not observe SVC1 bacteria on H&E‐stained sections in any tissue sample, suggesting that the bacteria were rapidly cleared and/or that invasion was rapid and robust. Taken together, these results establish SVC1 as a safe bacterial delivery vehicle for repeated intranasal delivery to the respiratory tissues.

### 3.4 SVC1 is minimally immunogenic in the respiratory tract

**FIGURE 4:**
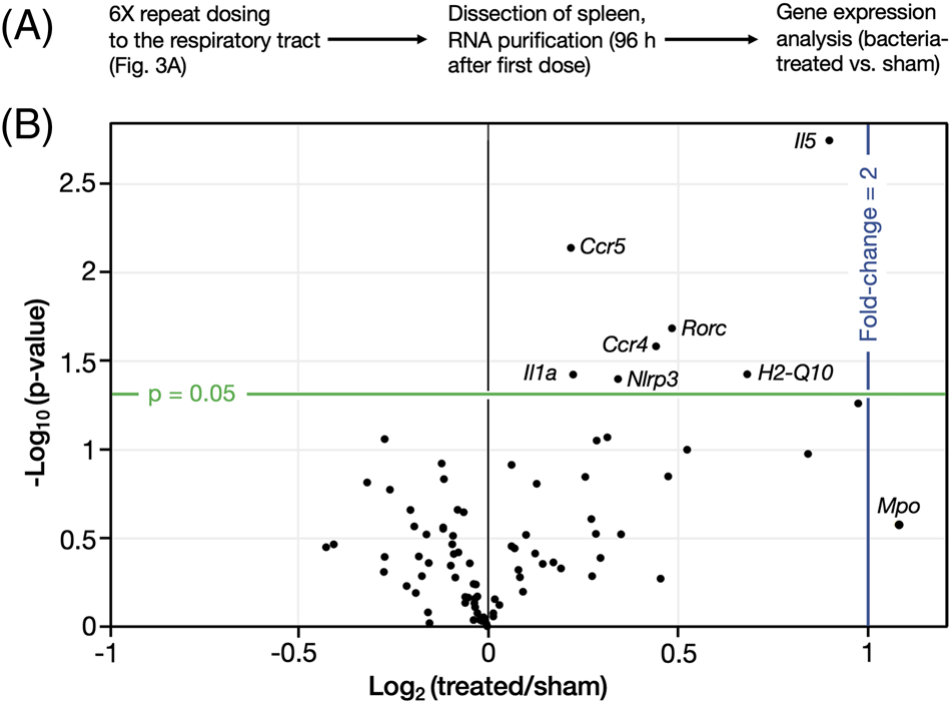
Repeated in vivo dosing of invasive bacteria to the lungs is elicits a negligible immune response. (A) Mice were dosed with invasive SVC1 bacteria or sham as in Figure 3. The spleens were removed 24 h after administration of the last dose, and RNA was isolated for analysis using the Qiagen RT2 Profiler Mouse Innate and Adaptive Immune Response PCR Array. (B) A volcano plot of differential gene expression after 6× dosing with invasive bacteria versus sham treatment (PBS). Expression differences were considered statistically significant at *p* > 0.05 (green line). A fold‐change of 2 is indicated by the blue line. Genes with statistically significant expression level differences or a fold‐change greater than 2 (i.e., *Mpo*) are labeled. The difference for *Mpo* was not statistically significant

To evaluate the systemic immunogenicity of SVC1 in the respiratory tract, we collected the spleens from the mice in the repeat dosing experiment, purified total RNA, and examined gene expression differences between sham and SVC1‐treated mice using a Qiagen RT^2^ Profiler PCR Array for mouse adaptive and innate immune responses (Figure 4A). This array allows simultaneous monitoring of 84 immune‐related genes, which taken together are representative of key innate and adaptive immune responses. As SVC1 was developed from a commensal, highly attenuated *E. coli* strain, we expected that it would be minimally immunogenic in the respiratory tract, even after repeated dosing. Among the 84 immune‐related genes analyzed, only seven had statistically significant (*α* = 0.05) expression level changes in the SVC1 bacteria‐treated mice compared with the sham‐treated mice: *Ccr4*, *Ccr5*, *IL1a* (Interleukin 1a), *H2‐Q10*, *Il5* (interleukin 5), *Nlrp3*, and *Rorc* (Figure 4B). The upregulation of these genes likely reflects the presence of bacteria, as each has been linked to responses to bacterial lipopolysaccharide (LPS) [58, 59, 60, 61, 62, 63, 64, 65]. The upregulation of LPS‐related genes is not unexpected because while the LPS of SVC1 is truncated (lacking the O‐antigen), it remains recognizable, though only mildly immunogenic, in mammals [66, 67, 68]. In addition, the gene encoding myeloperoxidase (MPO) was upregulated over 2‐fold. While this change was not statistically significant, it could also reflect an effect of bacterial presence as MPO has been linked to a response to pro‐inflammatory agents in the lung epithelium [69]. Repeated treatment did not result in significant non‐specific or specific changes in the expression levels of pattern recognition receptors (PRRs), cytokines/chemokines, innate/adaptive markers, inflammatory or bacterial defense markers. Taken together, these data indicate that repeat dosing with SVC1 to the respiratory tract in mice does not induce a robust immune response compared to PBS (sham)‐dosed mice, suggesting that SVC1 stimulates a negligible immune response and is safe for repeat dosing in a mammal.

### 3.5 SVC1 can deliver anti‐viral shRNAs to the respiratory tract in vivo

**FIGURE 5:**
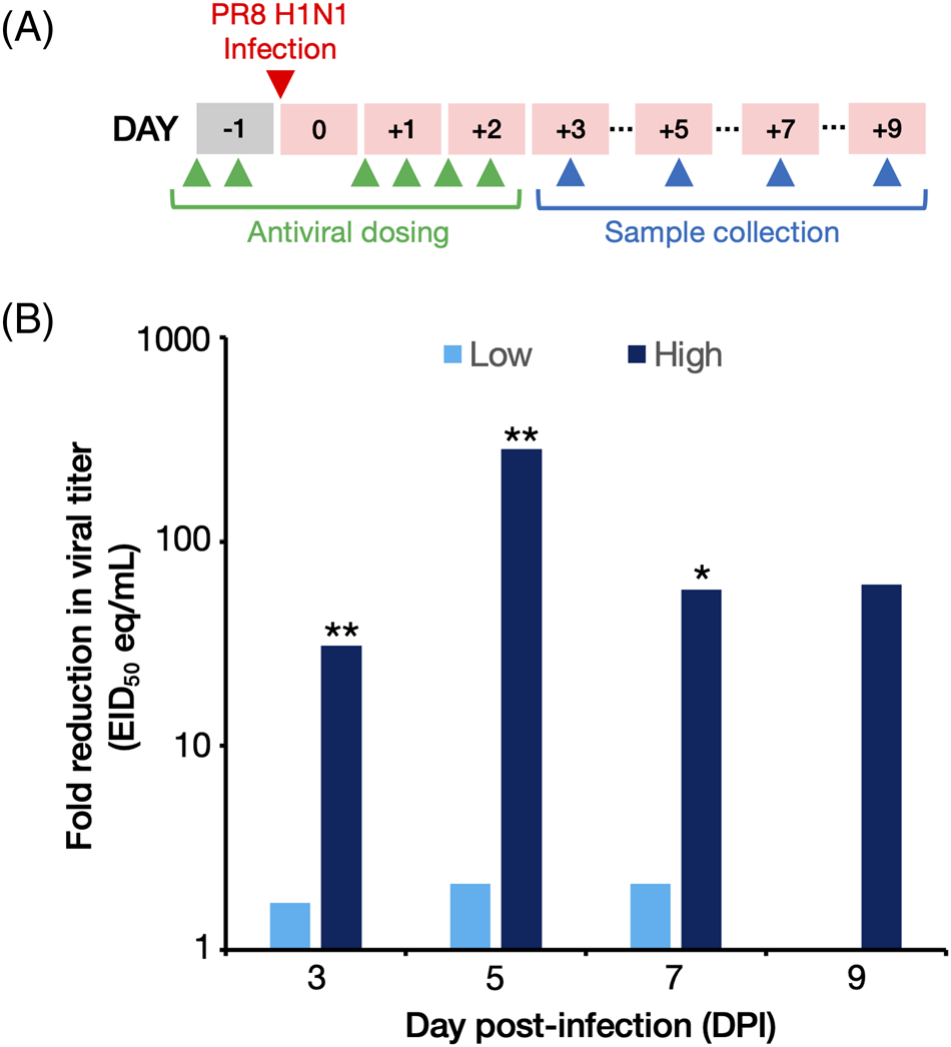
Bacterially delivered influenza‐targeting shRNAs mitigate influenza virus replication in vivo. (A) Mice (*n* = 6 per group) were treated with high (1 × 108 CFU/mL) or low (1 × 106 CFU/mL) doses of bacteria expressing influenza‐targeting shRNAs or PBS sham twice prior to and four times after (green arrows) infection with PR8 (H1N1) influenza virus (red arrow). Samples of nasal turbinate tissue were collected for assessment of viral titer up to nine days post‐infection. (B) Plot showing the fold reduction in viral titer (as EID50 eq/mL, see Section 2) in mice dosed with shRNA‐expressing bacteria or PBS (sham) from 3 to 9 days post‐infection (DPI). Statistical significance was calculated using the two‐sample Wilcoxon rank‐sum (Mann–Whitney *U*) test. Asterisks indicate statistical significance between sham‐treated (control) and SiVEC‐IAV‐treated animals: *, *p* ≤ 0.05; **, *p* ≤ 0.01

Finally, we wanted to demonstrate the in vivo therapeutic potential of SVC1 to deliver antiviral shRNAs. To this end, we designed two therapeutic strains: an SVC1 derivative expressing an shRNA against the influenza A virus (IAV) PA protein (RNA polymerase complex subunit) (SVC1‐PA) and an SVC1 derivative expressing an shRNA against the influenza NP protein (nucleocapsid) (SVC1‐NP). These strains are mixed prior to administration to produce the SiVEC‐IAV cocktail. Upon SiVEC‐IAV administration, the shRNAs are delivered to the cytoplasm of respiratory epithelial cells (the site of IAV replication), and they are processed via the RNAi pathway into siRNAs that silence PA and NP expression, thereby inhibiting IAV replication and reducing viral shedding. To test the efficacy of these shRNAs delivered via SVC1, we dosed mice with a cocktail of SVC1‐PA and SVC1‐NP at two concentrations (low and high) twice prior to viral challenge and then four times after the mice were exposed to H1N1 IAV (PR8 strain) as described in Figure 5A. On days 3, 5, 7, and 9 post‐challenge, we collected the nasal turbinates, purified total RNA, and determined the viral titers (as EID_50_ equivalent/mL) via reverse transcription quantitative PCR (RT‐qPCR). As shown in Figure 5B, reductions in viral titer were observed at the low and high bacterial doses, with a clear dose–response trend. As expected, the high dose was the most effective at reducing viral titer in the nasal turbinates. These results demonstrate that SVC1 can be used as an effective vehicle for the delivery of therapeutic shRNAs to the lungs in a respiratory disease model.

## 4 DISCUSSIONS

A significant factor limiting the translation of therapeutic nucleic acids, proteins, and gene editing technologies from bench to bedside is the absence of safe and robust vehicles for targeted delivery to affected cells and tissues. In the case of nucleic acids, this limitation is imposed by the negative charge, instability, immunogenicity, and in some cases the large size, particularly relative to typical small‐molecule drugs. The SVC1‐based platform described here offers an elegant solution to the targeted delivery conundrum and holds promise for utility in ameliorating a range of diseases and disorders. SVC1 was engineered (i) to constitutively express nucleic acids, (ii) to target clinically relevant cell types (i.e., mucosal epithelial cells), and (iii) to escape the phagosome allowing the release of the nucleic acids into the target cell cytoplasm. SVC1 can be tailored to produce different types of therapeutically relevant nucleic acids. Furthermore, based on the targeted administration results shown in Figure 2, SVC1 might be useful for treating a plethora of oral, respiratory, gastrointestinal, ocular, vaginal, and rectal diseases. While not investigated here, SVC1 can be used for delivery of proteins, eukaryote‐translatable mRNA, and gene‐editing systems (e.g., CRISPR/Cas) to targeted mucosal tissues, providing cellular uptake and, where appropriate, nuclear translocation of the gene‐editing nuclease system, without the need for host genome integration.

A key feature of any drug delivery system is the amount of therapeutic moiety that can be delivered to the targeted cells. In human cells, siRNAs are not enzymatically replicated to allow transgenerational inheritance of the silencing effect, in contrast to the well‐known transgenerational inheritance of RNAi in nematodes [70]; therefore, delivered shRNAs are diluted during cell division as the finite supply of molecules is depleted upon partitioning to daughter cells. While it was not technically feasible to measure the number of shRNAs delivered by SVC1 bacteria at the time this work was completed, the persistence of the silencing effect shown in Figure 1D provides hints to understanding this key feature of the system. The unexpected findings that the silencing effect of the shRNAs produced and delivered by SVC1 were sustained for at least 96 h post‐treatment, a time period sufficient for the initially invaded cells to divide [71, 72], suggests that SVC1 cells deliver a large payload of shRNAs, attesting to the potential for a sustained therapeutic effect. Importantly, this effect is achieved without genome integration and is ultimately transient as the populations of the delivered shRNAs and processed siRNAs are depleted either via dilution upon cell division or due to the inherent instability of RNA molecules (the latter being especially relevant to the transiency of the effect in post‐mitotic cells).

The intracellular delivery modalities currently in use in the clinic, for example, lipid nanoparticles and viral vectors, suffer from undesirable immune effects that can limit their utility, particularly for repeated dosing. The preliminary analysis of the immunogenicity of SVC1 delivered to the lungs (after six repeated doses over 3 days) presented here suggests that the bacteria are minimally immunogenic, as no significant immune cell infiltration (Figure 3F) or statistically significant changes in the expression level of any screened immune‐related gene (2‐fold or greater) (Figure 4) were observed. Interestingly, of the systemically upregulated genes detected after repeated respiratory administration, genes related to cellular responses to LPS were overrepresented (seven out of seven) [58, 59, 60, 61, 62, 63, 64, 65]. This observation suggests that the array analysis used here is sufficiently sensitive to detect subtle gene expression changes in response to the presence of the bacterial LPS but that the amount of LPS delivered via this administration scheme was insufficient to induce a robust systemic immune response. Finally, tissue damage was not observed even in tissue directly exposed to the bacterial vehicle (Figure 3B–E). We are currently further modifying SVC1 to express a less‐immunogenic LPS to further mitigate immunogenicity concerns that may arise with increasing dose or systemic administration. Work is also underway to explore whether the SVC1 system is affected by acquired immunity. However, an acquired immune response to the SVC1 bacteria is not anticipated based on the gene expression analysis described here as well as the additional genetic modifications we are making to further attenuate the LPS of SVC1.

To establish the potential of SVC1 as an in vivo therapeutic delivery vehicle, we demonstrated that simultaneous delivery of shRNAs designed to silence two essential influenza genes (the SiVEC‐IAV cocktail) could ameliorate viral replication in a mouse model of influenza infection (Figure 5). To our knowledge, this work represents the first demonstration of the use of a bacteria‐based delivery system in a mammalian antiviral application. The robust reductions in viral replication shown in Figure 5B confirm that SVC1 can indeed be used to deliver therapeutic RNA molecules. Importantly, our data also revealed a dose‐dependent reduction in viral replication when different numbers of SVC1 cells were intranasally administered. This dose responsiveness demonstrates that the number of bacteria delivered can be modulated to achieve different therapeutic outcomes, which might be advantageous in some applications of such a platform (e.g., delivery of gene editing components).

Finally, a distinguishing advantage of the SVC1 bacterial delivery platform (in comparison to other available delivery platforms) is that the bacteria themselves can produce the therapeutic moieties that they deliver, as demonstrated here by the bacterial transcription of shRNAs that feed into the host cellular RNAi pathway. This feature of the system eliminates RNA manufacturing steps and production costs [73, 74, 75, 76]. As it is simple and fast to generate large quantities of bacteria using widely available manufacturing approaches, SVC1‐based therapeutic products could be readily generated in massive quantities from a small stock, and if properly stored, could have a long shelf life. With manufacturing in mind, we are currently working on characterization of potency, including developing methods to quantitate the number of shRNA molecules generated per SVC1 bacterial cell.

Ongoing research efforts are underway to further develop nucleic acid delivery and targeting as well as bacterial delivery systems [81, 83, 84, 85]. We are currently optimizing SVC1 as a platform for the production (via bacterial transcription) and delivery of both linear and circular eukaryote‐translatable mRNAs and for the production and delivery of gene editing proteins and RNAs (i.e., CRISPR/Cas machinery). We are also actively engaged in evaluating the platform for systemic delivery via intravenous injection in a mouse model, and our preliminary data show that SVC1 can be safely administered via tail vein injection. This outcome was expected based on the modifications we have made to the bacterial LPS and other safety features. We expect that the bacteria will have broad tissue distribution following systemic administration, allowing it to be useful for applications requiring broad tissue delivery. As β1 integrin is expressed on many types of cancer cells, work is currently also underway to use SVC1 to deliver anti‐tumorigenic therapeutics (nucleic acids and proteins) to cancer cells, both in vitro and in vivo. Due to the vast genetic coding capacity and transcriptional flexibility of *E. coli*, SVC1 can express and deliver high molecular weight RNA molecules. Furthermore, our deep knowledge of *E. coli* molecular genetics enables further application‐specific optimization (e.g., additional modulation of RNase activities) to improve its performance as a highly versatile delivery platform. We expect that the advantages offered by live bacteria, and SVC1 in particular, will lead to future studies that further enable and validate the usefulness of bacteria as a powerful multi‐application delivery platform.

## Supporting information

SUPPORTING INFORMATION

## Data Availability

The data that support the findings of this study are available from the corresponding author upon reasonable request.
