# Supplementary material for: An optimized live bacterial delivery vehicle safely and efficaciously delivers bacterially transcribed therapeutic nucleic acids
Source: Eng Life Sci. 2023 Feb 5;23(3):e2200037. doi: 10.1002/elsc.202200037 (PMC9978928; doi:10.1002/elsc.202200037)
Supplement: Supplementary file 1 — SUPPORTING INFORMATION [file ELSC-23-e2200037-s001.pdf]

Supporting Information

An optimized live bacterial delivery vehicle safely and efficaciously delivers bacterially transcribed therapeutic nucleic acids

Mora et al.

A549 cells + SVC1/pE2-Crimson

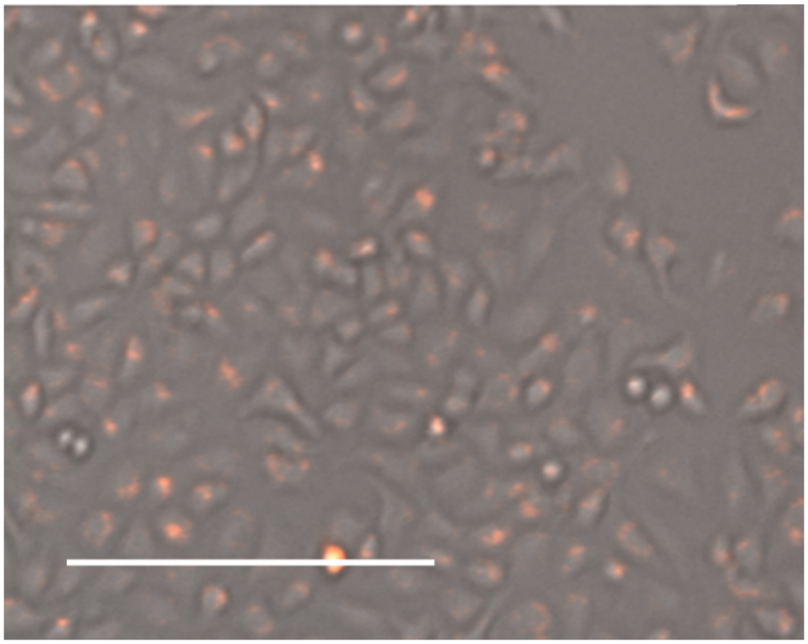

Supplemental Figure 1. Invasion of A549 cells by SVC1 bacteria expressing red fluorescent protein. Scale bar represents 0.25 mm.

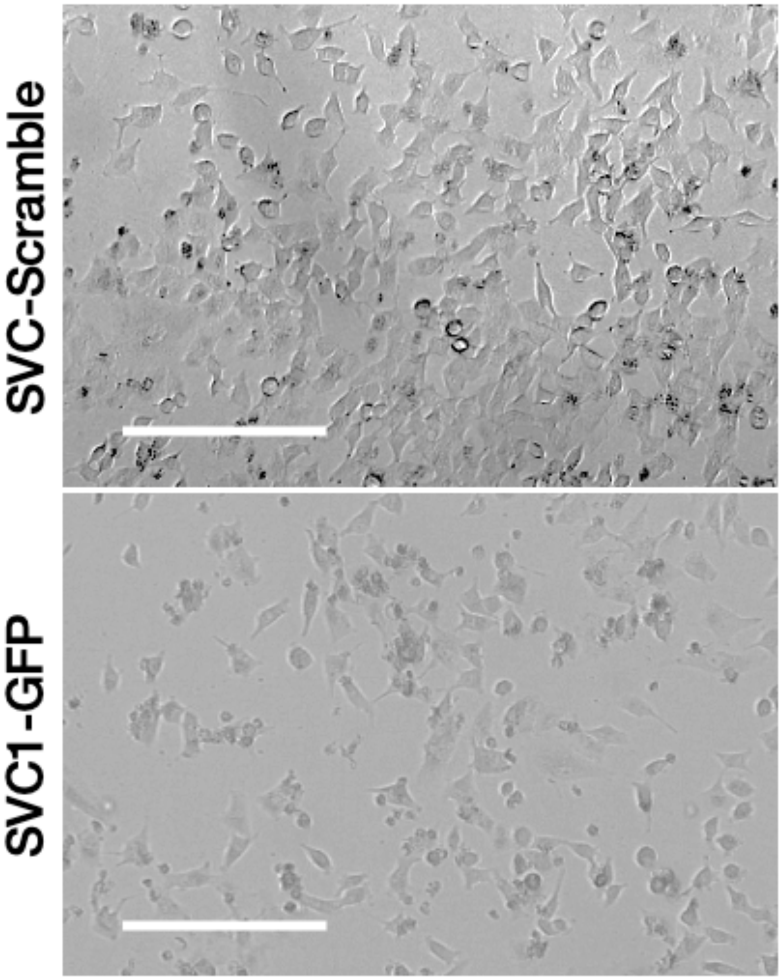

Supplemental Figure 2. Brightfield channel for the fluorescent images shown in Figure 1. Scale bar represents 0.5 mm.

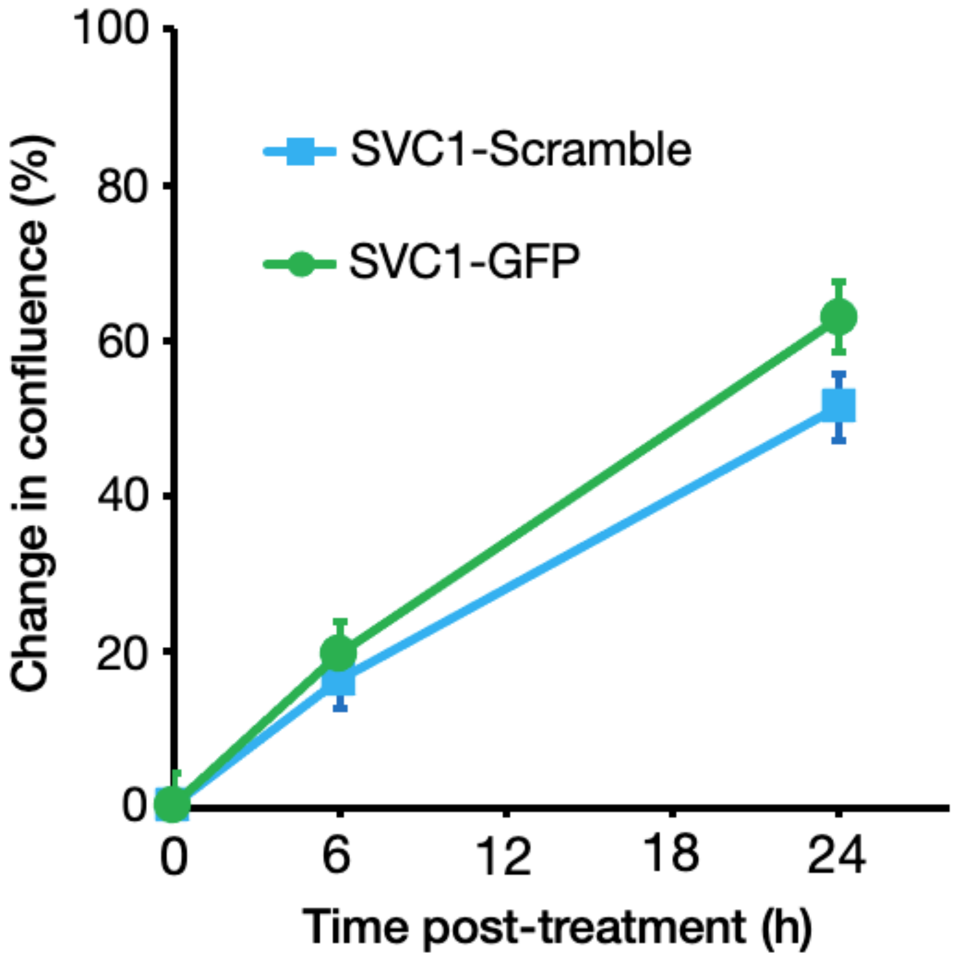

Supplemental Figure 3. Proliferation of A549 cells under the growth conditions described in the Materials and Methods. Plot shows the mean ± SD at each time point.
